# A basic study on molecular hydrogen (H_2_) inhalation in acute cerebral ischemia patients for safety check with physiological parameters and measurement of blood H_2_ level

**DOI:** 10.1186/2045-9912-2-21

**Published:** 2012-08-23

**Authors:** Hirohisa Ono, Yoji Nishijima, Naoto Adachi, Masaki Sakamoto, Yohei Kudo, Kumi Kaneko, Atsunori Nakao, Takashi Imaoka

**Affiliations:** 1 Department of Neurosurgery, Nishijima Hospital, Oooka, Numazu City, Sizuoka, Japan; 2 Department of Surgery, University of Pittsburgh, Pittsburgh, USA

## Abstract

**Background:**

In animal experiments, use of molecular hydrogen ( H_2_) has been regarded as quite safe and effective, showing benefits in multiple pathological conditions such as ischemia-reperfusion injury of the brain, heart, kidney and transplanted tissues, traumatic and surgical injury of the brain and spinal cord, inflammation of intestine and lung , degenerative striatonigral tissue and also in many other situations. However, since cerebral ischemia patients are in old age group, the safety information needs to be confirmed. For the feasibility of H_2_ treatment in these patients, delivery of H_2_ by inhalation method needs to be checked for consistency.

**Methods:**

Hydrogen concentration (HC) in the arterial and venous blood was measured by gas chromatography on 3 patients, before, during and after 4% (case 1) and 3% (case2,3) H_2_ gas inhalation with simultaneous monitoring of physiological parameters. For a consistency study, HC in the venous blood of 10 patients were obtained on multiple occasions at the end of 30-min H_2_ inhalation treatment.

**Results:**

The HC gradually reached a plateau level in 20 min after H_2_ inhalation in the blood, which was equivalent to the level reported by animal experiments. The HC rapidly decreased to 10% of the plateau level in about 6 min and 18 min in arterial and venous blood, respectively after H_2_ inhalation was discontinued. Physiological parameters on these 3 patients were essentially unchanged by use of hydrogen. The consistency study of 10 patients showed the HC at the end of 30-min inhalation treatment was quite variable but the inconsistency improved with more attention and encouragement.

**Conclusion:**

H_2_ inhalation of at least 3% concentration for 30 min delivered enough HC, equivalent to the animal experiment levels, in the blood without compromising the safety. However, the consistency of H_2_ delivery by inhalation needs to be improved.

## Introduction

Molecular hydrogen (H_2_) is the most abundant element in the universe but the concentration in the air at ground level is very low (0.00006 volume %) [1]. In human, however, intestinal bacteria produce a large quantity of H_2_[2] and it was a common belief that the implied sufficiency and possibly continuous availability precluded any beneficial effects of the internal H_2_ on human health, except perhaps in some anecdotal miracle healing water stories. Recently, however, extensive research on medical H_2_ in the areas of cell biology, animal disease models and others have introduced irrefutable evidences [3,4] that medical treatment with H_2_ is feasible on human diseases. However, since H_2_ exists in the close environment and in human body itself, it cannot be therapeutic to human disease unless it reaches the diseased areas much closer or directly. In that sense, delivery by the blood or vascular system from the intestine or from externally administered H_2_ to the medically needed sites is essential. It is also unlikely to be therapeutic if tissue H_2_ concentration (HC) after external H_2_ administration remains at the same range as HC of the naturally occurring H_2_. At least, the therapeutic tissue HC needs to reach the blood or tissue HC level reported by animal experiments [2]. In addition, the level should be achieved consistently each time with the external H_2_ administered for the treatment. In clinical medicine, it cannot be usable unless the therapeutic HC level causes no aggravation of physiological parameters and no detrimental changes in patient’s conditions.

Since the expertise in our hospital includes treatments of ischemic cerebral disease, we are most interested in neuroprotective effects of H_2_ and have treated some of these patients with H_2_ after successful trials with volunteers, as has been reported elsewhere. We concluded at that time that H_2_ treatment, when it was combined with other medications, provided some visible benefits to a very selective and small number of brainstem infarction patients [4].

In the present study, we intended to share basic data from our clinical experience with particular attention to the above mentioned essential factors for clinical application of H_2_ treatment. HC in the arterial and venous blood during and after H_2_ gas inhalation was measured with gas chromatography. For the safety evaluation, a complete set of physiological parameters was observed serially before, during and after H_2_ administration and at the same time with measurement of HC in the arterial and venous blood. Although some of these data are already available in the animal experiments, we felt an additional study was needed since most of our cerebral ischemia patients belong to high age group, who have limited ability to cope with any detrimental effects of a new therapy. We also evaluated the consistency of our H_2_ delivery methods by measuring the HC in the venous blood at the end of 30- min H_2_ treatment on 10 patients in multiple occasions. We believe that these data are essential not only for the safety of the patients but also for objective interpretation of effects of H_2_ treatment.

## Materials and methods

Thirteen patients with acute ischemic cerebral disease were involved in this study. All of the participants were provided with sufficient information regarding H_2_ administration and signed a consent form which had been approved by the Nishijima hospital ethics committee, Nishijima hospital pharmacy committee and also use of the hydrogen product in the hospital had been conducted by the Nishijima Hospital Pharmacists Council and the Tokai Hokuriku District Burrow of Japanese Welfare-Labor Administration and the Pharmaceutical Affair (Regulatory Audit Section) of the Sizuoka Prefectural Administration. Before recruiting the patients to the current study, a complete PARQ conference was given to all of the patients and their family and the nature of the study was explained to them. They perfectly understood that the H_2_ treatment may not provide any benefit to the patient’s condition but may help better understanding of the nature of the hydrogen treatment and may promote hydrogen research for the future and further development of cerebral ischemia treatment with hydrogen. They were also told that the study will be discontinued immediately if there occurs any sign of side effect and/or excessive discomfort and/or any other reasons for them to stop the hydrogen treatment.

### Production and administration of H_2_- enriched intravenous fluid and H_2_ gas

H_2_- enriched intravenous solution was produced by simply immersing the intravenous fluid bags in the hydrogen water tank (Miz.Co, Patent No.4486157, Patent Gazette of Japan 2010) as has been reported elsewhere [5]. H_2_ gets in the bag by diffusion through the bag wall. Although H_2_ concentration in the water tank was at the saturation (0.8 mM), the H_2_ concentration in the bag varied with the duration of immersion and with the material of the bag wall and by the method of infusion, as reported elsewhere [6]. Essentially, approximately 90% of the original concentration measured at the tip of the infusion catheter remained at the end of 30 min infusion. H_2_ gas for inhalation was prepared with an apparatus, made in our hospital by Yoji Nishijima MD, using a H_2_ generator (HG200, GL Science, Tokyo, Japan), a small air compressor and an oxygen inlet from the wall oxygen outlet unit. An appropriate concentration of H_2_ gas was mixed with the air and additional oxygen as needed. The gas product was provided to the patient through a mixed gas reservoir (a transparent plastic bag) and a regular facial mask. The patients inhaled the gas by their own effort and speed but for the patients who were on mechanical ventilation, the gas was given through the respirator. The apparatus and the set up with a regular facial mask were shown to the patients and their family with detailed explanation before their signing of the consent form.

### Measurement of hydrogen concentration (HC) in the blood

A 72 year old person (case 1) who has been comatose and on respiratory support inhaled 4% H_2_ gas through the ventilator and case 2 (78 year old) and case 3 (74 year old) inhaled 3% H_2_ gas through a facial mask. Ten blood samples were taken from existing blood access ports at 10, 15, 20, 30, 40, 42, 46, 52 min and 58 min after the administration of H_2_ and a control blood sample was taken immediately before the study. The blood sample was placed in a 12 ml glass bottle immediately for the gas chromatographic measurement of HC. These 12 ml glass bottles were initially filled with fresh air in preparation and the top was secured with a hard plastic plug and additional screw top. The arterial or venous blood sample was injected in the bottle by briefly unscrewing the top. The blood sample consisted of 2 ml of arterial or venous blood withdrawn into a heparinized syringe, and 1 ml of the sample blood was put into the bottle and the rest was used for measurement of physiological parameters. Then, the bottle with the blood sample for HC check was placed in an ultrasonic cleaning device for 30 min vibration before the gas (vapor) in the bottle was aspirated and injected into a gas chromatograph device (TRIlyser, mBA-3000, Taiyo Co Ltd, Osaka, Japan).

### Measurement of physiological parameters associated with H_2_ administration

A set of the physiological parameters was studied immediately before, during and after completion of hydrogen treatment with inhalation on 3 patients as mentioned above. The set of the parameters included body temperature (BT), blood pressure (BP), pulse rate (PR), oxygen concentration related parameters (pO2 (Torr), sO2, pO2 (A-a), pO2 (a/A)). carbon dioxide related indices (pCO2 (Torr), HCO3-act (microM/L), and base excess related indices (microM/L), BE (ecf, concentration of titrable base of extracellular fluid), BE (B, titrable base of blood), BB (buffer base, total equivalent concentration of all the anionic buffering components).

### HC measurement for consistency study at the end of 30-min H_2_ administration

Ten acute cerebral ischemia patients participated in a short consistency study. Essentially, this was a hydrogen inhalation study where a disposable facial mask for regular oxygen inhalation was applied on these patients and a string attached to the facial mask was pulled to get a tighter fit. However, no dditional tape or other material was used to increase air tightness in order to avoid any discomfort to these old patients. The patients inhaled the mixed gas (3% hydrogen in the air) at their own effort and speed. At the end of 30-min treatment, a 2 ml venous blood sample was obtained from either existing IV port or by vein puncture and HC was measured by gas chromatography as above. Among these 10 patients, two patients had compromised pulmonary function from asthma and COPD (chronic obstructive pulmonary disease). When the result of HC measurement was reported to be very low in these two patients, the following inhalation was supplemented with slow intravenous infusion of hydrogen-enriched saline solution at the rate of 200cc /hr (or total of 100ml in 30 min) for concern of aggravating their pulmonary congestion. This speed of infusion was in the range of routine IV fluid administration but much slower than usual hydrogen-enriched saline administration in our previous study [5,6]. Some patients were excluded from the study immediately when the patient declined the venous puncture and/or complained of any discomfort/dissatisfaction.

## Results

### HC in the blood

**Figure 1:**
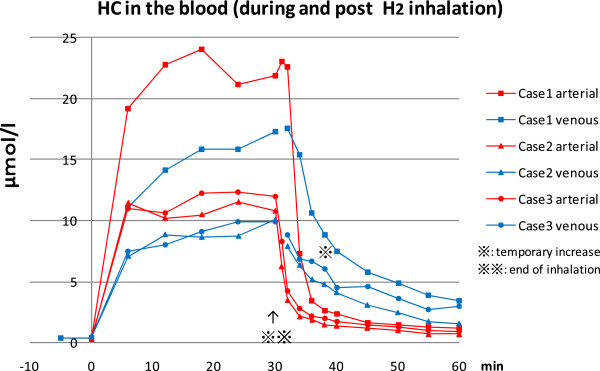
**Hydrogen concentration (HC) in the blood before, during and after hydrogen (H**_**2**_**) inhalation.** HC (micromol/L) in the arterial(red) and venous (blue) blood before (5 min. before), during (0-30min.) and after (30 min to 60 min) H_2_ gas inhalation. Case1 (square marks) inhaled 4% H_2_ gas and case 2 (triangle marks) and case 3 (round marks) inhaled 3% H_2_ gas. The HC at the plateau level reached the equivalent values reported in the successful animal experiments. Presence of a hump in the descending venous blood concentration curve may indicate the source of extra H_2,_ coming out of slow blood flow/slow release compartments such as muscle and skin.

HC in the blood increased rapidly both in the arterial and venous blood after the initiation of H_2_ inhalation with 3% (case 2, 3) and 4% (case 1) of H_2_ gas and reached a plateau at approximately 10 to 20 microM/L respectively, in about 20 min. The HC in the arterial blood was always higher than venous HC but the difference between the two decreased with time. When H_2_ administration was terminated, the arterial HC abruptly decreased to less than 10% of the pre-termination level in 6 to 8 min. On the other hand, the venous HC changed rather slowly and decreased to less than 50% of the pre-termination value in about 6 min and continued to decrease further down to less than 10% of the plateau level in about 18 min. However, the decrease appeared to be not steady and linear as compared to the arterial HC and frequently associated with flattening of the decreasing curve or even occasional temporary increase at around 8 to 9 min after the end of H_2_ inhalation. However, no sign of H_2_ accumulation is noted (Figure 1).

### Physiologic parameters during and after hydrogen inhalation

**Figure 2:**
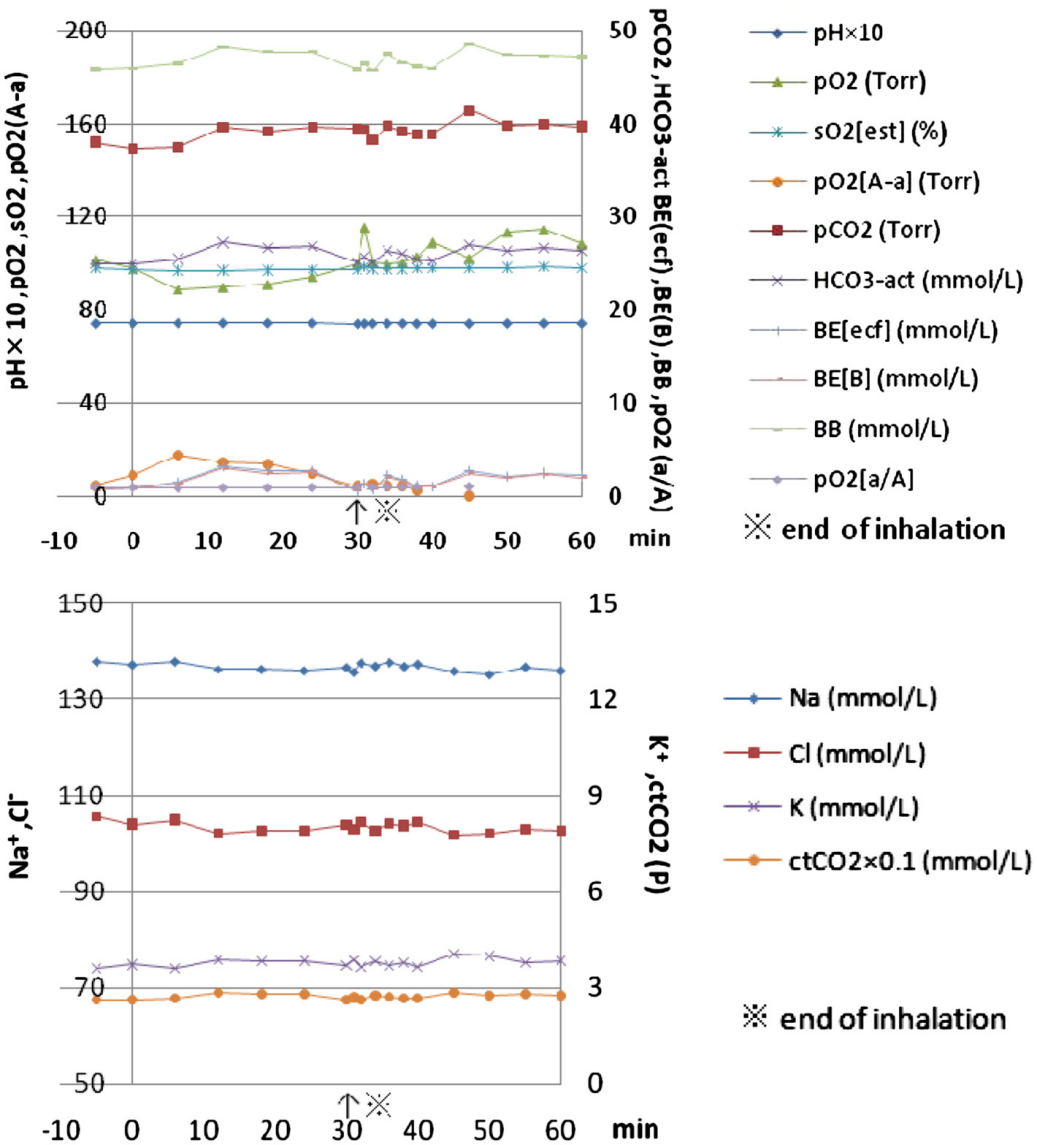
**Physiological parameters before, during and after Hydrogen (H**_**2**_**) inhalation.** Physiological parameters of case 1, before (5 min before), during (0-30min) and after (30 min to 60 min-) inhalation of H_2_ (4% in air), obtained simultaneously with the measurement of blood HC as in the Figure 1. These parameters and close clinical observation showed no significant changes except some indices related to respiration pattern such as hyperventilation or breath holding which were commonly seen among neurologically compromised patients with normal conscious level.

Most of the physiological parameters checked remained stable. Among these parameters, the most stable indices were pH and sO_2_ (oxygen saturation rate of the blood) in both arterial and venous blood (Figure 2). In the few unstable cases, the indices related to arterial PO_2_ and base excess related parameters such as BE and BB changed to a minor degree (less than 10%) during the inhalation. However, the pattern of these changes indicated that either hyperventilation or breath holding may be the cause, since these irregularities in the breath pattern were not rare when a face mask was unfamiliar or uncomfortable to the patients. However in earlier cases where H_2_ was added to the air up to 4% concentration, there was some indication that these changes may have been caused by a mild decrease (0.8 volume %) of O_2_ concentration in the inhaling hydrogen gas mix. The decrease in PO_2_ in the case 1 was associated with slight increase in base excess and pCO_2._ On the contrary, in case 2 and case 3 (not shown), the slight decrease in PO_2_ was associated with decreased pCO_2_ and BE indices, suggestive of hyperventilation. However, the slight decrease in PO_2_ of case 1 may reflect our earlier practice to mix H_2_ with air rather than air and additional oxygen. The change to add oxygen up to 40% concentration into the mixed gas reservoir was made to avoid any detrimental effect of low concentration of oxygen in the ischemic tissue.

### Consistency of HC at the end of 30-min hydrogen administration

**Figure 3:**
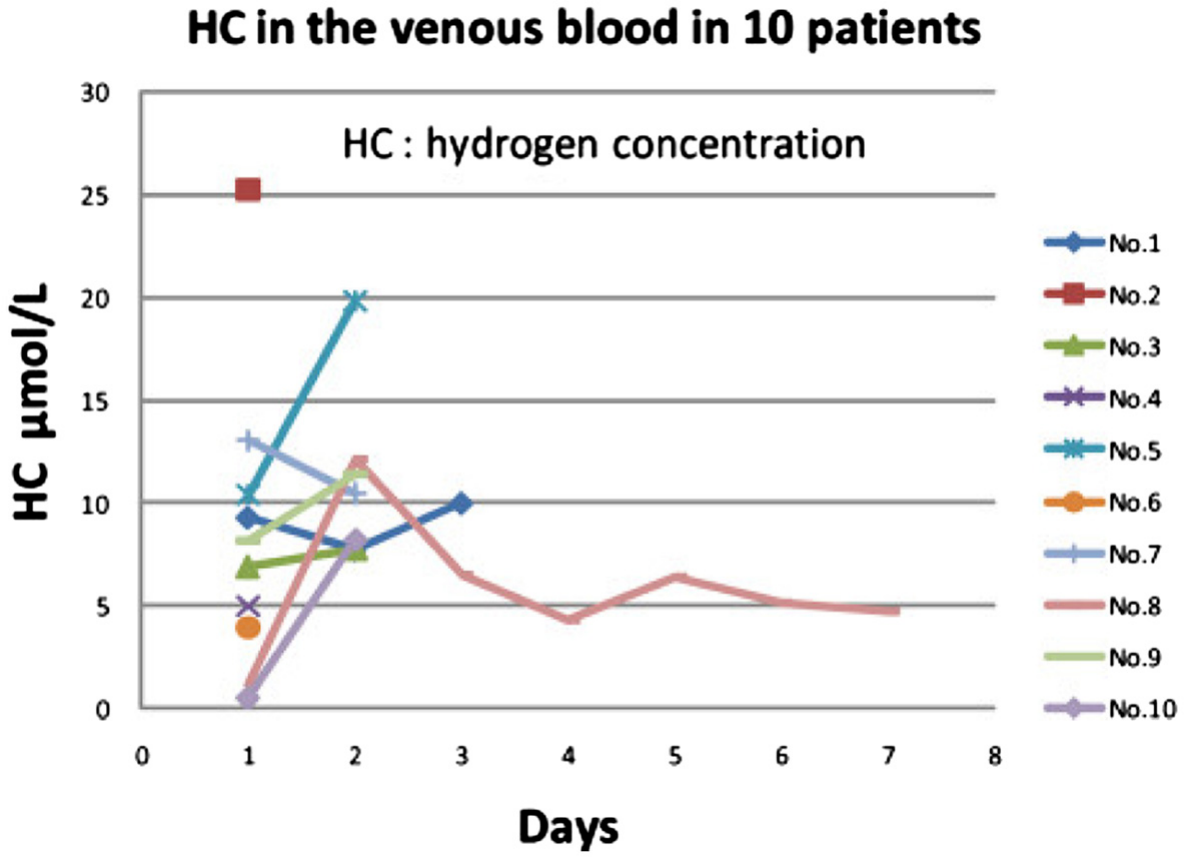
**Inconsistency of blood HC after initial H**_**2**_**inhalation and subsequent improvement with more attention.** HC in the venous blood varied widely, ranged from less than 1 microM to 25 microM/L on the first day of 30-min H_2_ inhalation. After closer observation at bedside and encouragement, the HC level and consistency improved. In two patients (Case No.8 and10) with pulmonary disease, the initial low HC significantly improved with simultaneous slow intravenous infusion of H_2_ enriched saline solution (from 1.2 to 12.1 microM/L in case No.8, from 0.5 to 8.2 microM/L in case No.10).

HC in the venous blood at the end of 30-min inhalation of the same mixed gas with 3% H_2_ concentration was not identical among the participated 10 patients and actually it varied widely, ranging from less than 1 to 25 microM /L (Figure 3). However, when these patients with low HC were watched more closely with frequent check of the facial mask and encouragement, the HC usually increased. In two patients with pulmonary disease (Case No.8 and No.10), the hydrogen inhalation raised the HC at the end of 30 min to less than 1 microM/L level on the first day, but addition of intravenous administration of hydrogen-enriched saline solution (100cc of H_2-enriched_ saline solution over 30 min period) simultaneously with inhalation, raised the venous HC significantly from 1.2 to 12.1 microM/L in the case No.8 and from 0.5 to 8.2 microM/L in the case No.10.

## Discussion

A large quantity of H_2_ gas is intestinally produced in human and the many animal experiments have shown some HC in the blood and tissue in a control condition without adding external H_2_[7-9].

However, in the current study, no HC in the blood was measurable by our method in the control condition where no external H_2_ was administered. The internal H_2_ is generated mainly in the colon by anaerobic bacteria during anaerobic metabolism as a pH dependent fermentation process [10]. Therefore, the amount of H_2_ production can be minimal in fasting and at rest as has been demonstrated both in animal experiments and in clinical cases [11]. Although the intestinal H_2_ production continues, the amount is reported to be only 1.6 ml/min. This can increase by 7-fold to 30-fold in various situations [1] but a large part of colonic H_2_ is dissipated by three main H_2_ consuming reactions (methanogenesis, sulfate reduction and acetogenesis) [12]. In addition, the residual H_2_ even at the maximum volume can be exhaled by the lung rapidly [13] because of very low blood solubility of hydrogen as compared to the air and a large gas exchange up to more than 6000 ml/min in the lung in human. The discrepancy in HC in control condition between our study and reported cases may be due to our patient’s NPO (nothing by mouth) status and/or bed rest, since healthy humans do not exhale H_2_ unless after food intake or in motion[1].

For the delivery of H_2_ to the medically needed area, H_2_ needs to be in the blood first. The presence of H_2_ was well demonstrated during H_2_ administration by inhalation in our cases and the level was much higher than the level associated with intravenous administration as reported previously [6]. This may suggest some advantage of H_2_ gas inhalation as compared to intravenous administration of H_2-enriched_ solution but the inconsistency associated with facial mask inhalation has to be solved first. In unattended patients particularly with neurologically compromised condition, the facial mask was frequently not on the appropriate position when our staff returned to stop the inhalation at the end of 30-min treatment. We found that a facial mask with a tightening belt around the head and face with a one way valve lessened the inconsistency but even a valve with a very light opening pressure caused sensation of respiratory obstruction in some patients. Use of respirator assistance and even a body position [14] may have to be considered for the consistency of inhalation treatment. However, use of a small, transparent mixed gas reservoir and very low one way pressure valve in our current set up significantly decreased the respiratory irregularity. Our current H_2_ mixed gas generator, made by Yoji Nishijima, M.D. was designed to provide capability of altering HC in the mixed gas reservoir at normobaric pressure, since the exact correlation between the HC in the inhaled gas and the blood level was not known at that time and also premixed H_2_ cylinder at high pressure, which appears to be more convenient, was initially not allowed to bring in the hospital for the fear of possible combustive accident. Intravenous administration of the H_2_- enriched IV fluid, on the other hand, appears to have practical advantages such as no need for inhalation delivery system, ease and relative comfort of the patients with more consistency, if more fluid can be given in a shorter time. However, our cerebral ischemic patients are usually in old age and many of them have some cardiopulmonary diseases and occasionally with kidney dysfunction. Fluid overload may have to be avoided on these patients. Therefore, the mode of H_2_ administration for a consistent delivery has to be determined by careful evaluation of general medical condition of each patient. Simultaneous administration of H_2_ inhalation and slow intravenous administration of hydrogen-enriched fluid may provide a better strategy in a difficult situation. A hasty and false conclusion that H_2_ was ineffective for the treatment can be easily drawn from faulty delivery without enough H_2_ in the diseased area. Therefore, a blood sample for HC at the end of inhalation may be important information not only for the consistency check but also for objective evaluation of H_2_ effect. It is also important not to mix H_2_ only with air in order to avoid possible hypoxia. Adding oxygen to the mixed gas certainly removes the possibility of tissue hypoxia and may provide some benefits of NBO (normobaric oxygen) treatment in the ischemic brain tissue as has been reported both in animal studies[15,16] and in clinical cases [17]. Although no conclusive evidence regarding benefit of NBO has been established yet and increased oxygen concentration in the cerebral ischemic focus may aggravate oxidative injury of the tissue [18], H_2_ immediately neutralizes hydroxyl radicals, the worst kind of the reactive oxygen species [3]. Therefore, NBO with H_2_ may be an ideal combination of medical gases for the treatment of cerebral ischemia, although the beneficial effects of the components may need to be evaluated separately. No significant change in the physiological parameters during and after H_2_ administration was noted except in some indices associated mainly with hyperventilation or breath holding. A majority of patients sensed no foreign smell and no subjective changes of any kind and their satisfaction was very high. In animal experiments also with H_2_ inhalation under more strict and defined condition, no change in the vital signs was seen. In addition to the proven safety, the total insensibility of any appreciable effects during and after H_2_ administration should qualify the H_2_ treatment as an ideal treatment regimen.

Our current study suffers multiple limitations. Firstly, the number of the patients recruited for the study was rather small and comparative data with other delivery methods, such as intravenous administration or ingestion of the H_2_- enriched fluid, are not sufficiently available. Since our study had a restrictive consent which required exclusion of the participant who complained of unexpected discomfort and/or dissatisfaction, prolonged data collection particularly for consistency study was difficult. A study with more stable and uniform design is required for determination of usefulness of facial mask for H_2_ administration. However, with improvements on the facial mask and inhalation technique, the inconsistency associated with H_2_ inhalation should be lower than ours.

## Competing interests

The authors declare that they have no competing interests and were not compensated at all by any pharmaceutical and biotechnology company to contribute this article to the peer-reviewed scientific literature.

## Authors’ contributions

The authors equally contributed to the production of this article except AN who provided editorial assistance. All authors read and approved the final manuscript.
